# Effect of intraoperative dexmedetomidine infusion during functional endoscopic sinus surgery: a prospective cohort study

**DOI:** 10.1186/s13037-020-00264-4

**Published:** 2020-10-13

**Authors:** Mohammad Reza Fazel, Zeynab Sadat Ahmadi, Hossein Akbari, Fahimeh Abam

**Affiliations:** 1 Department of Radiology, Faculty of Medicine, Kashan University of Medical Sciences, Kashan, Iran; 2 Research Center of Biochemistry and Nutrition in Metabolic Disorder, Kashan University of Medical Sciences, Kashan, Iran; 3 Department of Community Medicine, School of Medicine, Social Determinants of Health Research Center, Kashan University of Medical Sciences, Kashan, Iran

**Keywords:** Dexmedetomidine, Functional endoscopic sinus surgery, Pain, Blood loss

## Abstract

**Background:**

Dexmedetomidine, an α2 agonist, has well-known anesthetic and analgesic-sparing effects. We designed this study to evaluate the effect of intraoperative dexmedetomidine infusion on intra operative blood loss and postoperative pain in functional endoscopic sinus surgery.

**Methods:**

This prospective cohort study included 90 patients planning to undergo endoscopic sinus surgery, who were randomly divided into three groups. 2 to 2.5 mg/kg of propofol was used in all groups to induce anesthesia. One group received dexmedetomidine 0.2 μg/kg per hour infusion whereas the other group received dexmedetomidine 0.5 μg/kg per hour infusion. The control group received normal saline infusion.

**Results:**

The mean age of patients was 41.02 ± 11.93. 33 patients in the dexmedetomidine 0.2 μg/kg/h group, 30 patients in the dexmedetomidine 0.5 μg/kg/h group and 27 patients in the placebo group. The lowest amount of bleeding was related to the dexmedetomidine 0.5 μg/kg/h group. The volume of bleeding between the three groups was significantly different (*p* = 0.012). The satisfaction of the surgeon in the dexmedetomidine 0.5 μg/kg/h group was more than other groups. There was a significant relationship between the satisfaction of the surgeon and the treatment groups (*p* < 0.001). The lowest duration of surgery was related to the dexmedetomidine 0.2 μg/kg/h group. The most Trinitroglycerin (TNG) consumption was in the placebo group and the highest dose of labetalol was in the dexmedetomidine 0.5 μg/kg/h group. There was no significant difference in the TNG and labetalol consumption between three groups. The lowest consumption of morphine and pethidine in the dexmedetomidine 0.5 μg/kg/h group.

**Conclusions:**

Infusion of dexmedetomidine 0.5 μg/kg/h decreased blood loss and consumption of morphine and pethidine in patients who underwent endoscopic sinus surgery.

## Background

Rhinostenosis is an inflammatory process of the paranasal sinuses, which is diagnosed based on clinical manifestations, endoscopic findings, and changes in computed tomography (CT) scans [1].. The best treatment is endoscopic sinus surgery [2]. Bleeding is one of the most common problems during the sinus endoscopy. Chronic rupture disrupts the field of surgery and increases the risk of problems such as damage to the skull and eye cavity. Hemorrhage increases the duration of surgery due to several interruptions during surgery for suction and compression [3]. Postoperative bleeding also causes delay in discharge of the patient from the hospital [4]. Today, various techniques for reducing intra operative blood loss and the desirability of surgical field are used, which include: 1. Using an intravenous anesthetic method instead of anesthesia with inhalable gases, which has been identified in several studies that this method effectively reduces bleeding and improving the surgical procedure [5, 6]. 2. Use of local vasoconstrictor drugs, such as adrenaline [7], which is effective in reducing intra operative blood loss. 3- Use of anti-fibrinolytic drugs such as tranexamic acid. In several studies, it has been used as an additive [8], topical [8] and intravenous [9] for endoscopic sinus surgery, which has been effective in reducing bleeding and improving the field of surgery in all three methods. 4-Using deliberated hypotension with various drugs including trinitroglycerin and captopril [10] sodium nitroprusside [7] alpha-2 adrenergic agonists such as clonidine, dexmedetomidine, [4] which have been studied in this study to reduce bleeding and improve surgical operations. The above methods can have potential complications. Vascular contraceptive drugs may cause hemodynamic instability, especially in patients with a history of hypertension or ischemic heart disease [9]. Also, inhalation anesthetics cause delay in recovery [4]. And Sodium nitroprusside also causes tachyphylaxis and cyanide poisoning [4]. Recently, alpha-2 agonists have been used to control hemodynamics during surgery and have used them for analgesia in many surgeries. Dexmedetomidine is a highly specific and potent agonist of alpha-2 receptors that has analgesic, antinociceptive and anti-anesthetic properties, as well as sympathetic properties, which simultaneously does not result in respiratory suppression [4]. The central and peripheral sympathetic activity of dexmedetomidine, which is carried out by α2-adrenergic receptors, reduces arterial blood pressure and heart rate and releases norepinephrine dose-dependent. Considering the better effects of dexmedetomidine on hemodynamic control than Clonidine [11, 12], we decided to study the effect of dexmedetomidine on the volume of bleeding and the severity of postoperative pain in endoscopic sinus surgery.

## Methods

Current study is a prospective cohort study in ASA class I and II patients aged 15–75 years old and hemoglobin higher than 10 mg / dL who referred to Matini Hospital in Kashan, with pansinusitis undergoing endoscopic surgery of the nasal sinus, were studied in 2017.

Patients were examined by the otolaryngologist at the clinic and diagnosed with chronic sinusitis based on clinical observations and CT scan findings. After obtaining written consent, patients were randomly assigned to three groups using a randomized block table [13–17]. Patients in the first group received dexmedetomidine infusion at a dose of 2. μg/kg Patients in the second group received dexmedetomidine infusion at a dose of 0.5. μg/kg patients in the third group receive normal saline as a placebo. All surgical procedures were performed by a surgeon who did not have any information on the drug used by the patient. Also, the person who controlled the patient during surgery was unaware of the type of medication. On the day of surgery, patients underwent pulse oximeter monitoring, non-invasive blood pressure measurement, and a coronary artery electrocardiogram. An intravenous route was established for them. The patients received 10 ml/kg Ringer. Premedication was performed with midazolam 0.03 mg/kg and 2 mg/kg Fentanyl. Anesthesia induction was performed by propofol 2 mg/kg and used to ease intraperitoneal intubation of 0.5 mg/kg atracurium.

Patients with intubate tube size were fitted with age and sex, and infusion of propofol 100 μg/kg/min with 50% N_2_O and oxygen was used to continue anesthesia. During the operation, fentanyl 1 μg/kg and atracurium 0.15 mg/kg every 45 min were repeated. Mechanical ventilation was the same for all patients with a flow volume of 10 ml / kg, respiratory rate of 10 times per minute. At the end of the procedure, all patients with Atropine 30 μg/kg and Neostigmine 60 μg/kg were reversed. Systolic and diastolic blood pressure, heart rate, and saturated oxygen before and after induction, after intubation during surgery, were recorded every 15 min until the end of surgery. In the course of operation, if the field of bleeding was greater than Grade 3 (Table 1), to improve the surgical status of patients infusion of Trinitroglycerin (TNG) (0.25–0.5 g/kg/min) was started to reduce the mean arterial pressure (MAP) less than 80 mmHg. In patients whose blood pressure was not controlled by TNG or tachycardia, labetalol was used at doses of 5 mg. The amount of bleeding was calculated based on the lost volume and pharyngeal weight estimation. After the surgery and extubation, the patient was transferred to recovery. The patient was kept in recovery for 2 h. Demographic data of patients including sex, height and weight were recorded in a pre-designed questionnaire. Also, the length of surgery and anesthesia, hemoglobin before and 12 h after operation, the amount of TNG consumed, the amount of bleeding during operation and up to 12 h after surgery, the surgeon’s satisfaction with the operation was also noted.

**Table 1:** Mean and standard deviation of volume of hemorrhage and hemoglobin at arrival in the studied groups

Variable	Dexmedetomidine 0.2 μg/kg/h	Dexmedetomidine 0.5 μg/kg/h	Placebo	***p***-value*
**Hemorrhage volume (ml)**	252.7 ± 115	238.3 ± 87.7	312.9 ± 83.9	0.012
**Initial Hemoglobin(g/dl)**	13.90 ± 1.66	14.33 ± 1.70	13.94 ± 1.23	0.5

*ANOVA, *P*-values < 0.05 were considered statistically significant

### Statistical analysis

Initially, the normalization of the dependent variables is investigated using Kolmogorov Smirnov test [18–20], then, if the data are normal, binary variations in each group are performed using paired t-test and comparison of the two groups is done using independent t-test [21–27]. Also, Wilcoxon rank-sum test and Mann–Whitney U test were used to analyze abnormal data. Finally, analysis of variance with repeated measurements was used to analyze the changes of related factors over time**.** *P*-values < 0.05 were considered statistically significant [28–31]. All statistical analyses were done using the Statistical Package for Social Science version 19 (SPSS Inc., Chicago, Illinois, USA) [32–34].

## Results

In this study, 90 patients were studied. The mean age of patients was 41.02 ± 11.93. 33 patients in the dexmedetomidine group 0.2 μg/kg/h, 30 patients in the dexmedetomidine group received 0.5 μg/kg/hr. and 27 patients in the placebo group. Sexual abnormalities did not differ significantly between the three groups.

In Table 1, based on the results, the lowest volume of hemorrhage associated with the dexmedetomidine group is 0.5 μg / kg / h. The volume of bleeding was significantly different between the three groups (*p* = 0.122). The LSD post hoc test showed a significant difference between the control and the other two groups, while the difference between the two concentrations of dexmedetomidine was not observed (*p* < 0.05). Hemoglobin was not significantly different between the three groups at baseline (*p* = 0.50).

Also, the results showed that the satisfaction of the surgeon in the dexmedetomidine group was 0.5% μg/kg/h more than other groups and there was a significant relationship between the satisfaction of the surgeon and the treatment groups (*p* < 0.001).

As shown in Table 2, the lowest pain levels at 2, 6, 12 and 24 h after surgery were related to the dexmedetomidine group 0.5 μg/kg/h. The analysis of variance with repeated measurements showed the effect of time on pain intensity changes, which indicates changes in pain intensity in the three studied groups during the measurement times (*P* < 0.001). Also, the interactive effect of time and therapeutic groups on pain changes revealed the difference between treatment groups over time on pain changes (*P* = 0.013).

**Table 2:** Mean and standard deviation of pain intensity in the studied groups at different times

Time	Dexmedetomidine 0.2 μg/kg/h (*n* = 33)	Dexmedetomidine 0.5 μg/kg/h (*n* = 30)	Placebo (*n* = 27)	*P	**P	***P
**2 h after surgery**	2.66 ± 1.67	2.13 ± 1.45	4.77 ± 1.16	> 0.001	> 0.001	0.013
**6 h after surgery**	3.18 ± 1.62	2.23 ± 1.47	4.44 ± 1.64	> 0.001		
**12 h after surgery**	2.54 ± 1.60	1.70 ± 1.41	3.40 ± 1.47	> 0.001		
**24 h after surgery**	1.96 ± 1.26	1.33 ± 1.15	2.59 ± 1.24	> 0.001		

*ANOVA ** time effect*** time groups effect. *P*-values < 0.05 were considered statistically significant

Also, according to the results, the lowest heart rate before induction, after induction and end of operation with dexmedetomidine group was 0.5 μg/kg/h, but in the recovery phase it was 0.2 μg/kg/h. Analysis of variance with repeated measurements revealed the effect of time factor on heart rate changes, which indicates changes in heart rate in the three studied groups during measurement times (*P* < 0.001). Also, there was no interaction between time factor and therapeutic groups on changes in heart rate, which indicates that there is no difference between treatment groups over time on changes in heart rate(*P* = .392).

In Table 3, based on the results, the lowest systolic blood pressure before the induction was related to the 0.2 dexmedetomidine group, after the induction and the end of operation was related to the placebo group, and in the recovery state the dexmedetomidine group was 0.5. Analysis of variance with repeated measurements revealed the effect of time factor on systolic blood pressure changes, indicating changes in systolic blood pressure in the three studied groups over time (*P* < 0.001). The effect of time agent and therapeutic groups on the changes in systolic blood pressure was also seen, indicating the difference between treatment groups over time on systolic blood pressure changes (*P* = 0.024).

**Table 3:** Mean and standard deviation of systolic blood pressure in the studied groups at different times

Time	Dexmedetomidine 0.2 μg/kg/h (*n =* 33)	Dexmedetomidine 0.5 μg/kg/h (*n =* 30)	Placebo (*n =* 27)	*P	**P	***P
Before induction	125.39 ± 18.23	129.50 ± 13.21	126.29 ± 20.59	0.630	> 0.001	0.024
After induction	125.84 ± 17.32	124.63 ± 15.03	122.14 ± 19.70	0.710		
End of surgery	110.48 ± 16.74	113.43 ± 15.73	105.48 ± 9.62	0.124		
Recovery state	111.27 ± 13.24	109.26 ± 10.97	118.25 ± 12.52	0.019		

* ANOVA ** time effect*** time groups effect. *P*-values < 0.05 were considered statistically significant

In Table 4, the least amount of diastolic blood pressure was observed before and after the induction and the end of the operation in the placebo group. In the recovery, the lowest pressure was related to 0.2 dexmedetomidine. Analysis of variance with repeated measurements revealed the effect of time factor on diastolic blood pressure changes, which indicates diastolic blood pressure changes in the three groups studied during the measurement times (*P* < 0.001). Also, the interaction effect of time agent and therapeutic groups on diastolic blood pressure changes, which indicates the difference between treatment groups over time on diastolic blood pressure changes (*P* = 0.013).

**Table 4:** Mean and standard deviation of diastolic blood pressure in the studied groups at different times

Time	Dexmedetomidine 0.2 μg/kg/h (*n =* 33)	Dexmedetomidine 0.5 μg/kg/h (*n =* 30)	Placebo (*n =* 27)	*P	**P	***P
Before induction	77.24 ± 9.75	82.60 ± 8.85	76.74 ± 8.40	0.026	> 0.001	0.013
After induction	78.36 ± 7.92	80.20 ± 10.01	73.03 ± 11.46	0.020		
End of surgery	72.18 ± 12.31	74.96 ± 12.34	68.74 ± 11.27	0.155		
Recovery state	70.15 ± 10.10	70.40 ± 12.37	75.18 ± 9.76	0.148		

*ANOVA ** time effect*** time groups effect. *P*-values < 0.05 were considered statistically significant

Based on the results, the highest percentage of arterial oxygen saturation (SPO2) before and after recovery was related to the placebo group. After induction was related to 0.5dexmedetomidine group and at the end of operation the dexmedetomidine group was 0.2. Analysis of variance with repeated measurements showed the effect of time factor on SPO2 variations, which indicates that SPO2 changes were not observed in the three study groups during the measurement times (*P* = 0.386). Also, the interaction effect of time agent and treatment groups on SPO2 changes was observed, indicating no difference between treatment groups over time on SPO2 changes (*P* = 0.523).

In Table 5, the duration of the operation and the amount of blood pressure reducing drugs used in the studied groups at different times are presented. The lowest duration of action was related to the 0.2 dexmedetomidine group. LSD post hoc test showed a significant difference between the control and the other two groups. However, this difference was not observed between the two concentrations of dexmedetomidine (*p* < 0.05). The highest TNG consumption was in the placebo group and the highest dose of Labetalol was in the 0.5 dexmedetomidine group. There was no significant difference in TNG and Labetalol in the three groups (*P* = 0.401).

**Table 5:** Duration of operation, and the amount used drugs for reducing blood pressure in the studied groups at different times

Time	Dexmedetomidine 0.2 μg/kg/h (***n =*** 33)	Dexmedetomidine 0.5 μg/kg/h (***n =*** 30)	Placebo (***n =*** 27)	***p***-value*
**Surgery duration (min)**	74.54 ± 13.99	79.83 ± 21.99	85.55 ± 13.03	0.047
**TNG**	2.01 ± 2.50	1.98 ± 2.24	2.24 ± 1.88	0.896
**Labetalol**	11.96 ± 8.47	15.16 ± 11.02	13.14 ± 8.45	0.401

*ANOVA. *P*-values < 0.05 were considered statistically significant

In Table 6, the mean and standard deviation of drug consumption in the studied groups are presented at different times. As it can be seen, the lowest levels of morphine and pethidine used were related to the 0.5 dexmedmotidine group.

**Table 6:** Mean and standard deviation of drug consumption in the studied groups at different times

Time	Dexmedetomidine 0.2 μg/kg/h (*n =* 33)	Dexmedetomidine 0.5 μg/kg/h (*n =* 30)	Placebo (*n =* 27)	***p***-value*
**Morphine (mg)**	1.24 ± 1.75	1.00 ± 1.25	1.62 ± 2.27	0.414
**Pethidine (mg)**	9.51 ± 11.00	3.00 ± 7.02	14.88 ± 10.77	> 0.001

*ANOVA. *P*-values < 0.05 were considered statistically significant

## Discussion

Overall, 90 consecutive patients were enrolled in this study.to determine the effect of dexmedetomidine on the volume of bleeding and the severity of postoperative pain in endoscopic sinus surgery. The mean age of patients was 41.02 ± 11.93 years. 33 patients in the dexmedetomidine group 0.2 μg/kg/h, 30 patients in the dexmedetomidine group received 0.5 μg/kg/h and 27 patients in the placebo group. The lowest amount of bleeding in the dexmedetomidine group was 0.5 μg/kg/h. The volume of bleeding between the three groups was significantly different (*p* = 0.122). The satisfaction of the surgeon in the dexmedmotidine group was 0.5% μg / kg / h more than other groups. The satisfaction of the surgeon was significantly correlated with the treatment groups (*p* < 0.001). The lowest pain levels at 2,6,12 and 24 h after surgery were related to the dexmedmotidine group 0.5 μg/kg/h. The lowest duration of action was related to the dexmedmotidine group 0.2 μg/kg/h. The LSD post hoc test showed a significant difference between the control and the other two groups, while the difference between the two concentrations of dexmedmotidine was not observed (*p* < 0.05). The highest TNG consumption was in the placebo group and the highest dose of Labetalol was 0.5 μg/kg/h in dexmedmotidine group.

In a study done by Anjan Das comparing the effect of dexmedmotidine and clonidine on controlled hypotension and postoperative endoscopic sinus surgery was shown that dexmedmotidine had a greater effect on the control of pressure and decreased bleeding during surgery and postoperative pain [11], which these findings were similar to those of our study.

In another study, the effect of dexmedmotidine versus Esmolol on controlled hypotension in endoscopic sinus surgery was investigated, which showed that dexmedmotidine was more effective [4]. In our study, the dexmedmotidine group in contrast to the placebo group also had more efficacy on controlling pressure and reducing bleeding during surgery and postoperative pain. The study by Alex Becker and colleagues found that the effects of dexmedmotidine and propofol doses compared to fentanyl and propofol on the quality of recovery and postoperative fatigue in patients undergoing spinal surgery revealed that recovery was better in dexmedmotidine-propofol group as well as patients had fewer postoperative symptoms [35], which is an indication of our study results. In another study, comparing the effect of dexmedmotidine versus placebo on recovery quality in 24 h after surgery, and its effect on postoperative vomiting, showed that single dose of dexmedmotidine improved recovery quality and decreased vomiting in recovery unit [36]. In our study, the group receiving dexmedmotidine had less relief in recovery compared to placebo. A study by Ramamani Mariappan. compared the effect of oral clonidine versus dexmedmotidine on the amount of need for anesthetic during surgery and the quality of recovery and hemodynamic control in spinal surgery, which showed that hemodynamic control and recovery quality in each two groups were equal and the amount of anesthetic was higher in the dexmedmotidine group [37]. In our study, the duration of the operation and the result of the anesthetic level in the dexmedmotidine group were less than 0.2 μg/kg/h of the other groups.

In another study, dexmedmotidine was compared with placebo on early postoperative cognitive impairment in postoperative laparoscopic cholecystectomy in elder people, which showed that dexmedmotidine significantly reduced postoperative early cognitive impairment in the elderly laparoscopic cholecystectomy surgery [38]. Other research group also examined the need for postoperative sedation drugs in the group of dexmedmotidine and total intravenous anesthesia (TIVA) versus Remifentanil and TIVA in Genomic Laparoscopic Video Surgery, indicating that the need for Postoperative analgesics for postoperative pain decreased in the dexmedmotidine and TIVA groups [39], which was similar to our study result. In another study, comparing the effect of dexmedmotidine+Desflurane and placebo+Desflurane on the amount of post-operative need of sedation drugs and postoperative vomiting and survival in the recovery unit in Laparoscopic bariatric surgery showed that the issues were decreased in the dexmedmotidine+Desflurane group, [40] which was similar to the result of our study.

## Conclusion

The lowest volume of bleeding related to the dexmedmotidine group was 0.5 μg/kg/h. The lowest pain levels in the 2,6,12 and 24 h postoperative pain group were 0.5 μg/kg/hr. dexmedmotidine. The lowest duration of action was related to the 0.2 dexmedmotidine group and the lowest levels of morphine and pethidine used were in the 0.5 dexmedmotidine group. Consequently, the current results realize that the infusion of dexmedetomidine reduces blood loss and consumption of morphine and pethidine in patients undergoing endoscopic sinus surgery.

## Data Availability

The dataset used in this study is available with the authors and can be made available upon request.
